# The Influence of Land Attachment on Land Abandonment from the Perspective of Generational Difference: Evidence from Sichuan Province, China

**DOI:** 10.3390/ijerph191811651

**Published:** 2022-09-15

**Authors:** Yue Zhang, Guihua Liu, Zhixing Ma, Xin Deng, Jiahao Song, Dingde Xu

**Affiliations:** 1 College of Management, Sichuan Agricultural University, Chengdu 611130, China; 2 College of Economics, Sichuan Agricultural University, Chengdu 611130, China; 3 Sichuan Center for Rural Development Research, College of Management, Sichuan Agricultural University, Chengdu 611130, China

**Keywords:** land attachment, land abandonment, generational differences

## Abstract

The deepening of rural population aging and the lack of labor transfer cause the phenomenon of land abandonment to become more serious, which threatens regional and even national food security. Based on the survey data of 540 farmers in Sichuan Province, the theoretical analysis framework of land attachment, intergenerational difference and land abandonment was constructed, and Probit and Tobit models were constructed to empirically analyze the influence of land attachment and intergenerational difference on land abandonment. Research results show that: (1) 10.9% of the farmers abandoned their arable land, with an average area of 0.17 mu; the interviewed farmers are mainly of the middle-aged generation; the scores of the three dimensions of farmers’ land attachment were all at the above average level. (2) Land dependence has no significant effect on land abandonment, while satisfaction and embeddedness have significant negative effects on land abandonment. (3) There are generational differences in the influence of land attachment on land abandonment. Among them, the land attachment of the middle-aged generation had no significant effect on land abandonment; the satisfaction and embeddedness of the older generation of farmers have negative effects on land abandonment; the satisfaction of the new-generation farmers has a significant negative effect on farmland abandonment. Based on this research, countermeasures and suggestions are put forward: (1) Pay attention to the emotional appeals of farmers and improve their well-being. (2) Cultivate new types of agricultural business entities and stimulate the potential of new human resources.

## 1. Introduction

With the rapid development of urbanization and industrialization, more and more of the young and middle-aged generations are going to cities to work. This large-scale labor migration has resulted in the loss of rural labor force and the decline of agricultural labor ability, and large areas of land are left uncultivated and abandoned [1]. According to the third National Land Survey, China’s arable land area is 1.918 billion mu, which has decreased by 113 million mu in the 10 years since the second National Land Survey. The abandonment of cultivated land is the main reason for the decrease in cultivated land, which affects China’s food security and agricultural and rural development. Today, the world is undergoing profound changes unseen in a century. The COVID-19 pandemic and the severe international situation are posing major challenges to food security. To this end, the 2022 No. 1 Central Document further stressed that the jobs of the 1.4 billion Chinese people should be firmly placed in their own hands at all times. If arable land abandonment continues and results in a large area of arable land abandonment across the country, we may be faced with a potential crisis of whether we can guarantee our food supply. In view of this, the problem of land abandonment should be paid enough attention and positive measures should be taken to deal with it.

At present, the related research in the field of land abandonment mainly focus on the spatio-temporal distribution characteristics, driving factors and its impacts. According to the international literature, since 1950, many developed and developing countries have seen the phenomenon of farmland abandonment [2]. Arable land abandonment is widely distributed [3], and the phenomenon of land abandonment in hilly and mountainous areas is particularly prominent [4,5]. The driving factors of land abandonment behavior have attracted much attention. Land abandonment is the result of the comprehensive effects of economy, system and nature [6,7]. From the perspective of the “rational man” in economics, most scholars believe that the main reasons for land abandonment lie in the low comparative income of agriculture, the high opportunity cost of rural labor and a large number of farmers voluntarily or forced to give up agricultural activities and migrate to cities [8]. At the level of the social system, the policy implementation level is low [9], the land transfer market is immature [10] and the transportation convenience degree is low [11], and the inadequate replacement function of institutional social insurance for land social insurance [12] and local ethnic conflicts [13] are also important reasons for the frequent occurrence of land abandonment in recent years. However, rural agricultural credit, especially informal agricultural credit [14], and the distribution of household Internet use in space and quantity [15] have an opposite aggregation trend with land abandonment. In addition, natural environmental factors, such as topography, soil type [16], land slope [17], vegetation destruction and climate change [18,19], also affect land abandonment. As for the influence of land abandonment, the academic circles mostly proceed from two aspects: food security and ecological environment effect. In terms of ecological effects, land abandonment is conducive to reducing disturbance caused by human activities [20], improving soil quality and fertility [21], and enriching vegetation diversity [22]. At the same time, it will also lead to a series of problems such as a decrease in biodiversity, an increase in fire frequency and intensity, and the loss of landscape and aesthetic values [23,24]. In terms of food security, most scholars believe that land abandonment reduces the cultivated land and the sown area of grain, which leads to the decline of grain output and reserve and the contradiction between supply and demand. Ultimately, it relies on imports and endangers food security. Of course, some scholars believe that land abandonment has both a negative feedback effect and positive significance on food stress [25]. In general, in the current shortage of per capita cultivated land and improper land use in China, the negative effects of land abandonment are still greater than its positive benefits. Therefore, it is necessary to deeply explore the driving factors of land abandonment and put forward targeted measures.

The research on the influencing factors of land abandonment has been quite rich, which provides some ideas for this paper. However, the existing literature mainly interprets land use behavior from the perspective of material economy and ignores the “irrational factor” of land attachment to a large extent. Human beings are the combination of “rationality” and “sensibility”, and almost all human activities are driven by emotional factors [26]. Farmers have a deep love and attachment to the land, that is, land attachment, which affects whether farmers are willing to abandon the cultivated land, and the depth of land attachment may directly affect the behavior and degree of abandoning the land. At the same time, farmers are the subject of family behavior decision-making, and their attitude, cognition and dependence on land directly affect land use [27]. Since China’s reform and opening up, with the rapid development of industrialization and urbanization, the heterogeneity among farmers has become prominent in terms of occupation and economic and social status. The heterogeneity of household groups constitutes household differentiation [28]. Household differentiation is one of the prominent phenomena in China’s rural areas, which affects the farmland utilization decisions of different households [29]. In essence, it is a process of farmers starting businesses away from the soil and splitting their land attachment [30,31,32]. This difference between farmers is reflected in time as generational change, and it is generational change that forms generational differences. However, the existing research not only lacks the discussion of the emotional factors behind the land abandonment, but also the “subjectivity” and internal differences of the farmer groups. On the basis of identifying the land attachment and intergenerational differences of the old and new generations of farmers, it is of great practical significance to explore the mechanism of land attachment on land abandonment.

In view of this, using the data of 540 households in Sichuan Province, this paper divided land abandonment into land abandonment behavior and land abandonment scale, and took intergenerational differences as moderating variables to explore the influence of land attachment on land abandonment, in order to provide a reference for alleviating the land abandonment phenomenon. The marginal contribution of this paper lies in the fact that: first, the theoretical analysis framework of land attachment, intergenerational difference and land abandonment is established, and the influence of land attachment and intergenerational difference on land abandonment is analyzed theoretically, which can provide a reference for similar research. Second, this paper empirically analyzes the influence of land attachment on land abandonment and analyzes the moderating mechanism of generational differences between them, which can guide the local practice of suppressing land abandonment more effectively.

## 2. Materials and Methods

### 2.1. Data Sources

**Figure 1:**
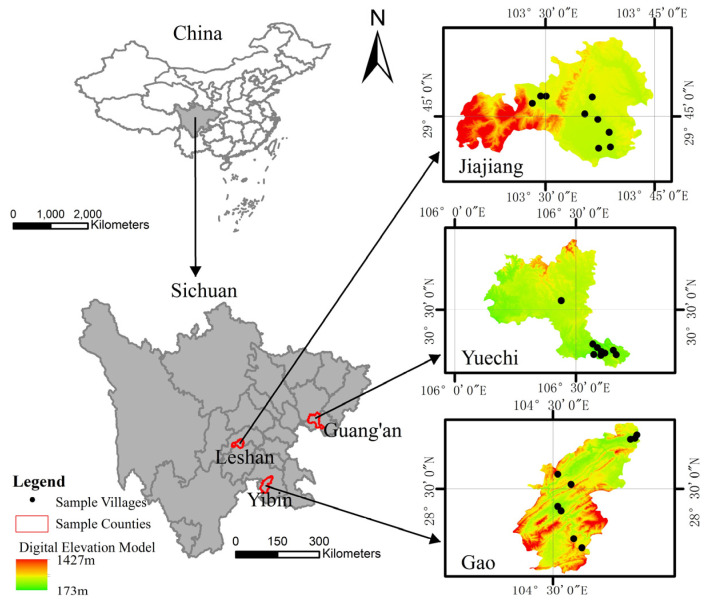
Spatial distribution map of the surveyed villages.

The data used in this research are from a survey in July 2021 in 9 towns and 27 villages of 3 counties in Sichuan Province, China. The questionnaire has a sufficient sample size and a wide range of research contents, mainly including basic information of farmers, capital status and land use. To ensure the representativeness of the sample, stratified equal probability sampling is adopted in this study. The specific steps are as follows: Firstly, according to the local economic development level, topography and other indicators, Jiajiang County (plain), Yuechi County (hilly) and Gao County (mountain) were selected as the research counties. Secondly, according to the distance between each township and the county government, the level of economic development and other indicators, three sample townships were randomly selected from each county. Then, three sample villages were selected from each sample village (Figure 1). Finally, 20 farmers were randomly selected from each sample village, and all the selected farmers or their family members were used as interviewees to conduct one-to-one interviews with the researchers. After the appeal investigation, 540 valid questionnaires were finally obtained. Among the 540 respondents, 59 farmers abandoned farmland, accounting for 10.9%; the average arable land abandoned by the 540 respondents was 0.17 mu. Among the farmers who abandoned their arable land, there were 20 farmers under 1 mu, 18 farmers at 1–2 mu, 8 farmers at 2–3 mu, 8 farmers at 3–4 mu, 4 farmers at 4–6 mu, and 1 farmer over 6 mu.

### 2.2. Theoretical Analysis and Research Assumptions

#### 2.2.1. Action Mechanism of Land Attachment on Land Abandonment

Place attachment theory has experienced the development and change of place theory, man–land relationship, love plot, place perception and place attachment. However, Chinese scholars’ research on land attachment is formed on the basis of western place attachment theory and combined with traditional Chinese farming civilization [33]. Place attachment refers to the emotional state in which people’s experience establishes a deep connection with a specific place. This positive emotional relationship is formed in a specific region over a long time, which is conducive to stimulating the emotional expression of farmers and effectively improving the willingness and behavior of community participation [34,35]. Land attachment can be said to be place attachment in a narrow sense [36]. It is the attitude of farmers to give land a special emotional and mysterious value, the condensation of all economic and emotional dependence of people on land, and an important window to see the evolution of the human–land relationship [30]. In terms of the measurement of land attachment, this paper refers to the research of Liu [36] and Xue et al. [37], and finally divides land attachment into three dimensions: satisfaction, embeddedness and land dependence. It includes three levels: farmer’s satisfaction with land use, attitude rooted in land, and economic and emotional dependence.

The traditional farming civilization continues to this day, which has had a profound impact on China’s grass-roots society. Land has always been the “food and clothing” of farmers, and land attachment is an emotion shared by all farmers. In general, the longer they have been exposed to farming civilization and the more opportunities they have to deal with land, the deeper their land attachment will be [38]. Farmers with a stronger land attachment will regard land as a treasure and have a stronger awareness of farmland protection but are not willing to abandon farmland. Farmers with weak land attachment consider more functional factors, but ignore emotional aspects, which may lead to the abandonment of some undesirable cultivated land. Most farmers with weak land attachment left the land and abandoned or transferred the land, and the land became the carrier of “homesickness” for countless wanderers.

**Figure 2:**
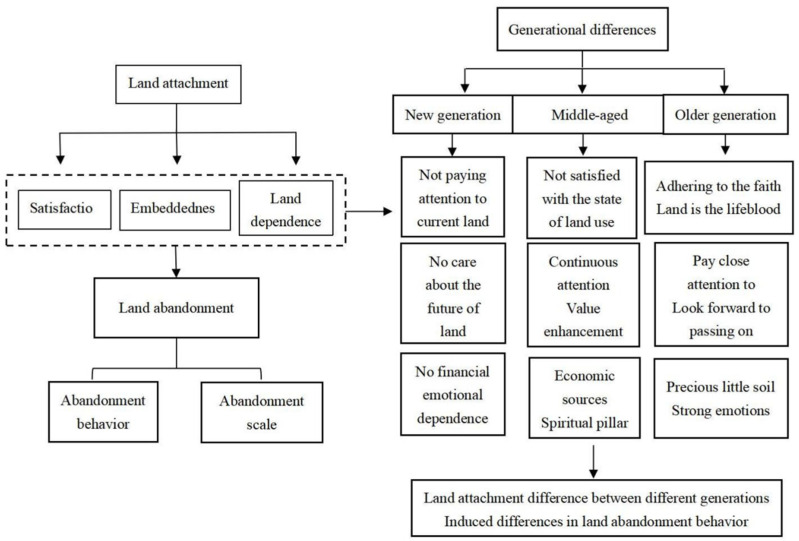
Conduction mechanism of intergenerational difference and land attachment on land abandonment.

Specifically, this dependence on and attachment to land is reflected in the economic and social security functions of land and the time, labor and emotional costs paid by farmers. From the perspective of satisfaction, embeddedness and land dependence, farmers who are more satisfied with the current state of land use will not choose to transfer out or abandon land because their input and output have reached a suitable balance and land resources have been effectively allocated. Farmers who are more deeply rooted in the land are always concerned about the land and hope that future generations will know about the land. They regard the land as a kind of blood inheritance and will not leave the land idle. Finally, farmers who are more dependent on land are not willing to abandon it because it has better economic and security functions and is also a good spiritual sustenance (Figure 2). Therefore, based on the above theoretical analysis, this paper puts forward the following hypotheses:

**Hypothesis** **1:** *Land attachment has a significant negative effect on farmers’ land abandonment.*

**Hypothesis** **2:** *Satisfaction, embeddedness and land dependence all have significant negative effects on farmers’ land abandonment.*

#### 2.2.2. Influence of Land Attachment on Land Abandonment from the Perspective of Generational Differences

Karl Mannheim was the first to study the differences between generations and to link “generation” to social change. Generation refers to groups born at the same time and experiencing the same social changes in the key growth stages, thus forming a collective identity [39,40]. Generational differences are considered to be the behavioral and cognitive differentiation between key groups of different generations caused by social environmental changes [41]. Groups in different generations have differences in values and preferences [39,42]. Therefore, with the development and changes of rural economy and society, there will inevitably be several generations of people with different attachment to land value.

Since China’s reform and opening up in 1978, the rapid development of urbanization and industrialization has not only brought about significant growth in economic level and improvement of international competitiveness, but also a profound impact on the evolution of rural man–land relationship [43]. In the process of urban–rural integration in China, with the rural market reform and the adjustment of agricultural industrial structure, the production behavior of farmers has changed from homogeneous pure farmers to heterogeneous non-farmers and part-time farmers, and the diversification of livelihood strategies [44,45] leads to the widening of the income gap between farmers. The boundary between the old generation of farmers and the new generation of farmers is increasingly clear [46]. At present, there is a phenomenon in China’s rural areas that “the post-1970s generation is unwilling to farm, the post-1980s generation will not farm, and the post-1990s generation will not talk about farming”. Different generations have different choices on the topic of “who will farm in the future”. In view of the differences in growth environment, personality characteristics, cognitive psychology and other aspects between farmers of different generations, the old generation of farmers and the new generation of farmers, have great differences in land attachment and urban identity, so there is a phenomenon of generational differences among farmers [43]: the new generation mostly left the land and their homes, and their land attachment was relatively weak. However, the middle-aged generation and older generations still maintained different degrees of attachment to the land after experiencing land changes and changes in their living environment, which further affected the arable land use behavior of different generations [41].

Overall, compared with the middle-aged generation and the new generation, the old generation of farmers is less educated and less employable, so they are more likely to stay in rural areas. In addition to the functional attributes of land economic security and the influence of many years of farming civilization, the older generation of farmers is more inseparable from land and has a stronger land attachment. Furthermore, they cherish land like gold and are unwilling to leave the land idle or transfer it. The middle-aged generations were either satisfied with the current situation or did not want to work anymore. If the land yield was good, they chose to transfer the land out or transfer it to others’ land to expand their operation [47], or they assumed the responsibility of “part-time farmers” under family pressure. Generally speaking, this generation has a strong land attachment and is more inclined to its economic value-added function, so usually less land is left idle. The new generations of farmers are generally better educated, can quickly adapt to the pace of urbanization, and yearn for urban life rather than staying in the countryside. As a result, most young people are indifferent to the land and generally give it to relatives and friends to manage, transfer or even abandon the land. It is precisely this difference in motivation and ability between generations that the labor force born in different generations has different emotional attitudes towards land, and then affects the use of farmland.

In terms of specific subdivision, the old generation of farmers have a belief in land, believing that “land is the lifeblood” and the cornerstone of their survival. They are highly satisfied with, rooted in and dependent on land, so they are not willing to abandon it. Driven by economic interests, most of the middle-aged generations were not satisfied with the current land use state, but they kept paying attention to land, taking land as an economic source and spiritual support. Most of the new generation of farmers leave their villages when they are young to start their own businesses. They pay less attention to the land and naturally have low satisfaction with the current land. They have no plans to return to their villages in the future and have almost no economic and emotional dependence on the land (Figure 2). Therefore, based on the above theoretical analysis, this paper puts forward the following hypotheses:

**Hypothesis** **3:** *The land attachment of the old generation and middle-aged generation inhibits the abandonment of land, while the land attachment of the new-generation farmers promotes the abandonment of land.*

**Hypothesis** **4:** *The satisfaction, embeddedness and land dependence of the older generation of farmers have negative effects on land abandonment; the satisfaction of the middle-aged generation significantly promoted land abandonment, while the embeddedness and land dependence significantly inhibited land abandonment; the satisfaction, embeddedness and land dependence of the new generation of farmers have a significant promoting effect on land abandonment.*

### 2.3. Variable Definitions

#### 2.3.1. Dependent Variables

It has been more than half a century since a large area of arable land was abandoned in the second half of the 20th century, but there is still no official unified definition of the connotation of “arable land abandonment”. Mantero et al. [48] believe that land abandonment means that human beings give up control of land (agricultural and forestry land, etc.), leaving land in a state of returning to nature. Keenleyside et al. [49] defines land abandonment as the land where all agricultural activities have stopped and plant replacement has begun. Gałecka-drozda and Zachariasz [50] made clear on this basis that if the abandonment time is 5 years or more, the land is considered abandoned. Based on Movahedi et al. [51], this paper defines land abandonment as a phenomenon that, under the joint action of some factors, farmers stop farming and other agricultural activities for a long time, resulting in idle and barren farmland.

The explained variables in this paper are the behavior and scale of land abandonment, which are obtained from the actual survey data. Among them, the behavior of abandoning land is measured by whether the household abandons land or not. If the answer is “yes”, the value is 1; otherwise, it is 0. The scale of land abandonment was measured by the actual land abandonment area (mu) of the household.

#### 2.3.2. Core Independent Variables

The core explanatory variable concerned in this paper is land attachment. Based on the ideas of Liu et al. [36] and Xue et al. [37], it is divided into three dimensions, with two indicators in each dimension for design. The answers to each indicator are divided into five levels: strongly disagree to strongly agree, and the variables are assigned a value of 1–5 according to the answers of farmers.

Among them, the first dimension is satisfaction, which measures farmers’ satisfaction with land use according to their identification degree of “I am satisfied with the current situation of land use” and “I am satisfied with the current situation of land use”. The second dimension is embeddedness. The questionnaire questions include “I hope my descendants can understand rural land” and “I always pay attention to my own land”, which mainly measures farmers’ sense of belongingness and attention to land. The third dimension is dependence on land. The questionnaire questions are “Land is not only the source of life for my family, but also my personal spiritual support” and “land is the basic guarantee of life, even if I go to the city to work or cannot engage in agricultural production”, which mainly measure the economic and emotional dependence of farmers on land. These three dimensions can depict the reality of rural farmers’ value judgment and emotional attachment to land.

#### 2.3.3. Adjusting Variables

Referring to the relevant literature, this paper selects the year of birth, namely age, to divide generations. Most scholars regard 1980 as the dividing line between the new generation of migrant workers and the first generation of migrant workers [52]. However, taking into account the fact that there is a certain lag in the formation of values in the “generation effect” and the serious aging phenomenon of the rural labor force [41], 1955 and 1970 are taken as the boundaries of generational division, thus forming dummy variables to reflect the differences between generations. If the head of the household was born after 1970, they are a new-generation farmer, and the value is 1. If they were born between 1955 and 1970, they are of the middle-aged generation, and the value is 2. If they were born before 1955, they are an older generation farmer, which is assigned a value of 3.

#### 2.3.4. Control Variables

With reference to studies by He Y et al. [5], Xu D et al. [53] and Shi T et al. [11], the following control variables are selected in this paper: the first is the characteristics of the household head, including the age of the household head and years of education; the second is the characteristics of the family, including the per capita annual income of the family and the proportion of non-agricultural income; third, the characteristics of the labor force, including the number of family labor force and the proportion of migrant labor force; fourth, the characteristics of land resources, including the per capita cultivated land area under management and the distance from home to market; fifth, social security features, controlling whether farmers pay pension insurance; and sixth, village characteristics, controlling village topography differences. In addition, considering that the economic development and functional area positioning of different locations will be different, which will directly affect the difference of farmers’ deserting behavior, the dummy variables of districts and counties should be controlled in the regression model. The description of relevant variables and descriptive statistics are shown in Table 1.

### 2.4. Research Methods and Models

#### 2.4.1. Probit Model

When the explained variable is “abandonment behavior”, it belongs to a dichotomous variable, so the Probit model was used to estimate benchmark regression, and the formula is set as follows:

(1) $$\;abandonment_{i}=h_{0}+h_{1}X_{1i}+h_{2}X_{2i}+h_{3}X_{3i}+h_{4}\sum CON_{i}+\varepsilon_{i}$$

In Equation (1), $\;abandonment_{i}$ indicates whether farmer I has abandoned land, $X_{1i}$ indicates the satisfaction of farmer *i*, $X_{2i}$ indicates the embedment of farmer *i*, $X_{3i}$ indicates the land dependence of farmer *i*, $\sum CON_{i}$ indicates the control variables, $h_{0}$ is the constant term, $h_{1}$, $h_{2}$ and $\;h_{3}\;$ represent the corresponding regression coefficients, and $\varepsilon_{i}$ is the random error term.

#### 2.4.2. Tobit Model

When the explained variable is “abandonment scale”, its value is approximately a continuous variable and there are many zero values. Therefore, the Tobit model is used to estimate the regression, and the model settings are as follows:

(2) $$\;abandonment\_sq_{i}=t_{0}+t_{1}X_{1i}+t_{2}X_{2i}+t_{3}X_{3i}+t_{4}\sum CON_{i}+\mu_{i}\;$$

In Equation (2), $\;abandonment\_sq_{i}$ represents the land abandonment scale of farmer *i*, and the settings of other variables are similar to those in Equation (1).

## 3. Empirical Results

### 3.1. Benchmark Regression Results of the Influence of Land Attachment on Land Abandonment

As shown in Table 2, the results of the benchmark regression show that farmers’ land attachment has dual effects on land abandonment. On the one hand, land dependence has no significant effect on land abandonment, probably because there were two conflicting forces: at present, the income of agricultural production is relatively low, and the dependence of farmers on the employment security function of land is weakened, so they will not choose to continue farming. However, due to the pension security function of land and the emotional dependence of farmers, they prefer to abandon the land rather than transfer out. On the other hand, both satisfaction and embeddedness had significant negative effects on land abandonment behavior and scale. Among them, satisfaction has a significant reverse effect on land abandonment at the 5% confidence level, and the abandonment behavior decreases by 3.3% and the scale decreases by 63.7% for each level of satisfaction increase. At the same time, the embeddedness had a significant negative effect on the abandonment behavior at the 10% confidence level, while it had a significant negative effect on the abandonment area at the 5% confidence level, and the behavior and scale of abandonment decreased by 2.3% and 38.5%, respectively. The reasons of satisfaction and grounding performance may be that: the farmers who are more satisfied with the current state of land use will not choose to transfer or abandon the land due to the effective allocation of land resources and the appropriate balance of input and output. The more deeply rooted farmers are in the land; they regard the land as a kind of blood inheritance and will not abandon the land. In short, the influence of land attachment on land abandonment is complex and changeable. There are not only factors promoting land abandonment, but also power sources hindering land abandonment, which mainly show an inhibitory effect. The regression results partially confirmed hypothesis 1.

### 3.2. Robustness Test of the Influence of Land Attachment on Cultivated Land

As shown in Table 3, in order to test the robustness of the above benchmark regression results, this paper selects the method of replacing the model for testing. Considering that the explained variable “abandonment behavior” is a dichotomous discrete variable, the Probit model is replaced by the Logit model to conduct a regression on the influence of land attachment on land abandonment. However, the “abandonment scale” is approximately a continuous variable, so the Tobit model is replaced by the Reg model. The regression results show that land dependence still has no significant effect on land abandonment. In contrast, satisfaction and embeddedness still had a significant negative impact on land abandonment at the 5% confidence level. This is basically consistent with the benchmark regression results, indicating that the results are robust and credible.

### 3.3. Intergenerational Differences in the Influence of Land Attachment on Land Abandonment

As for the specific impact of land plots of farmers of different generations on land abandonment, the regression results are shown in Table 4. The land attachment of the middle-aged generation has no significant effect on land abandonment, and it has an inhibitory effect on land abandonment. This indicates that for the middle-aged generation, the comparative income of agriculture was low, and land was not the object of their great concern. At the same time, the middle-aged generations were “rational” decision-makers to some extent and would choose to transfer or reuse their land instead of abandoning it. The satisfaction of the older generation of farmers and embeddedness both have inhibitory effects on land abandonment, and embeddedness has a significant negative impact on land abandonment at the 5% confidence level. This may be because the older generation has been dealing with land for a long time and believes that “land is the lifeblood” and the inheritance of blood. They have high expectations and attention on land, so they are not willing to abandon land. The satisfaction of the new generation of farmers has a significant inhibitory effect on land abandonment at the 5% confidence level. The reason may lie in the promotion of the current agricultural macro policy, the construction of the new agricultural management system, the cultivation of new professional farmers and the advocacy of moderate scale management, which make the young generation actively rush into rural construction and have high satisfaction with the current land, thus reducing the abandonment of arable land. However, the embeddedness and land dependence have certain promoting effects on land abandonment. This indicates that the growth environment of the new generation is different from that of the old generation, and it is not highly rooted and dependent on land, so there are also factors that promote its abandonment.

## 4. Discussions

Based on the survey data of farmers in Sichuan Province, this paper conducts a theoretical analysis on the influence of land attachment and intergenerational differences on land abandonment. The Probit model and Tobit model were constructed to empirically analyze the influence of land attachment and intergenerational differences on land abandonment. Its marginal contribution is: (1) To establish a theoretical analysis framework, and theoretically analyze the influence of land attachment and generational differences on land abandonment, so as to provide reference for similar research. (2) This paper empirically analyzes the influence of land attachment on land abandonment and analyzes the regulating mechanism of intergenerational differences in land attachment, which can more effectively guide the local practice of suppressing land abandonment.

The results show that, in the land attachment, satisfaction and embeddedness do have significant inhibitory effects on land abandonment, but the effect of land dependence is not significant, which partially confirms hypotheses 1 and 2. The reason for this inconsistency is that: The comparative income of land is low, the rural endowment insurance is further improved, and the economic dependence of farmers on land is weakened. However, at the same time, the emotional sustenance of the land makes the farmers unwilling to abandon the land. The two conflicts make the impact of household land dependence on land abandonment insignificant. Research suggests that the more connected farmers are to their land, the more likely they are to protect it [31,54]. Land attachment has a significant negative impact on land outflow, and land satisfaction, embeddedness and land dependence may have different impacts on land transfer [36], which is similar to the conclusion of this study. From the perspective of intergenerational differences, land attachment of the older generation did inhibit land abandonment, while land attachment of the middle-aged generation did not significantly affect land abandonment, which is consistent with hypothesis 4 that land attachment of the middle-aged generation had both promoting and inhibiting factors on land abandonment. The embeddedness of the new generation and land dependence do have a promoting effect on land abandonment, but it is not significant. Satisfaction has a significant effect on land abandonment, but it does not inhibit it, which is consistent with hypothesis 3 and 4. The reasons may lie in the promotion of agricultural macro policies, the construction of new agricultural management system, the cultivation of new professional farmers and the advocacy of moderate scale management, which make the young generation actively rush into rural construction and have high satisfaction and enthusiasm for the current land, thus having a certain buffer effect on reducing farmland abandoned. This conclusion is consistent with the conclusion of Cheng [27] in related research that the land attachment of the old generation of farmers is more important than rational income, the two are equivalent to the middle-aged generation, and the land attachment of the new generation is weaker than rational income. However, from the perspective of intergenerational differences, some studies have drawn the conclusion that the willingness and scale of land abandonment of the old generation of farmers are higher than the middle-aged generation and the new generation [41], which may be caused by the fact that this paper takes land attachment as the starting point and considers different factors from different perspectives.

There are two main deficiencies in this paper: (1) At present, this study only uses sectional data for empirical analysis, while panel data can better capture dynamic changes, which can be further explored in future studies. (2) The data in this study came from questionnaires of 540 households in Sichuan Province. Although sampling was conducted according to different landforms and landforms and different levels of economic development in plains, hills and mountains, whether it was applicable to other research areas remains to be studied. In view of the above deficiencies, we will continue to explore this in the future in order to have better development.

## 5. Conclusions and Policy Recommendations

Based on the survey data of 540 households in Sichuan Province, China, this paper constructed a model to analyze the influence of land attachment and intergenerational differences on land abandonment, and drew the following conclusions:

(1) Land dependence has no significant effect on land abandonment, while satisfaction and embeddedness have significant negative effects on land abandonment.
(2) There are generational differences in the influence of land attachment on land abandonment. Among them, the land attachment of the middle-aged generation had no significant effect on land abandonment. The satisfaction and embeddedness of the older generation of farmers have negative effects on land abandonment. The satisfaction of the new-generation farmers has a significant negative effect on land abandonment.

The above conclusions provide relevant policy implications for land protection policy:

(1) Pay attention to the emotional appeals of farmers and improve their well-being. Farmers are the main body of agricultural production, and farmers’ land attachment is an element that cannot be ignored in agricultural production. Especially for the old generation of farmers, although agricultural production is limited by labor force, their belief in the inheritance of blood and good expectation of land is also a kind of protection of land. For the middle-aged generation, they still retain a certain land attachment. At the same time, the main driver of rural labor flowing into cities is not the actual income gap, but the expected income gap. Therefore, while paying attention to farmers’ emotional appeals, retaining nostalgia and inheriting farming culture, we should also earnestly safeguard the dominant position of farmers, effectively protect their rights and improve their well-being. We will promote full cost insurance and planting income insurance for the three major grain crops to cover all major grain-producing provinces and major grain-producing counties, which will help stabilize farmers’ income expectations. Only by distributing one-time subsidies and other policy funds to farmers as soon as possible can we effectively play the main role of farmers and protect and mobilize their enthusiasm for growing grain.
(2) Cultivate new types of agricultural business entities and stimulate the potential of new human resources. In the face of the widespread phenomenon of household differentiation, it is particularly important to take measures with a definite target. The land attachment of different generations of farmers is different, which affects the decision of the family’s land use behavior. In particular, the new generation, which is strong and has a certain amount of capital, has a relatively weak land attachment, but its human resource endowment still has a great potential to play in the agricultural field. At present, the new professional management body is a major trend in the development of modern agriculture, which has created good conditions for the realization of large-scale, professional and mechanized management. We need to cultivate a new type of professional farmers who are literate, skilled in technology, good at business management, have a high sense of social responsibility and modern ideas, improve relevant welfare policies, and constantly enhance their ability to substitute crops and seeds. At the same time, it provides enough guarantees for farmers to go back to the countryside to work, agricultural materials to enter the village, agricultural machinery passage and agricultural technical personnel to sink, so as to reduce worries as much as possible. We will regularly check the grain area and the abandonment of arable land, constantly improve the guidance and service system for agricultural work and promptly find and solve problems.

## Figures and Tables

**Table 1:** Variable description and descriptive statistics.

The Variable Name	Variable Description and Assignment	Mean	SD
Abandonment behavior	Whether the household abandons land (yes = 1, No = 0)	0.109	0.312
Abandonment scale	Family abandoned area (mu)	0.174	0.677
Satisfaction	According to the average score of four dimension indicators	3.963	0.717
Embeddedness	According to the average score of four dimension indicators	4.073	0.767
Land dependence	According to the average score of four dimension indicators	3.707	0.843
Generational differences	Born after 1970 = 1, Born between 1955 and 1970 = 2, Born before 1955 = 3	2.137	0.713
Head gender	Age of Head of household (age)	58.93	11.02
Head education	Years of schooling of household head (years)	6.755	3.167
Gross annual income per capita	Annual household income/Family size (Yuan/person)	19,463	33,420
Non-agricultural income	Non-farm income/Total Household income (%)	0.752	0.298
Number of Labor force	Number of workers aged 16–64 (persons)	2.570	1.456
Proportion of migrant workers	Ratio of annual migrant workers to total family labor force (100%)	0.332	0.361
Per land area	Per capita land area under operation (mu/person)	1.434	4.257
Distance	Distance from home to market (km)	3.318	2.614
Insurance	Whether the family pays pension insurance (yes = 1, No = 0)	0.737	0.441
Village terrain	Mountain = 1, Hill = 2, Plain = 3	2	0.817

**Table 2:** Baseline regression of the influence of land attachment on land abandonment.

	Abandonment Behavior		Abandonment Scale	
	The Coefficient of *B*	*dy/dx*	The Coefficient of *B*	*dy/dx*
Satisfaction	−0.234 **	−0.033 **	−0.637 **	−0.637 **
	(0.104)	(−2.233)	(0.267)	(−2.386)
Embeddedness	−0.164 *	−0.023 **	−0.385 **	−0.385 **
	(0.086)	(−1.970)	(0.189)	(−2.044)
Land dependence	0.051	0.007	0.132	0.132
	(0.083)	(0.612)	(0.215)	(0.616)
Head age	0.001	0.000	0.032	0.032
	(0.020)	(0.034)	(0.047)	(0.690)
Head education	−0.018	−0.003	−0.011	−0.011
	(0.028)	(−0.627)	(0.077)	(−0.138)
Gross annual income per capita	0.098	0.014	0.104	0.104
	(0.137)	(0.740)	(0.360)	(0.289)
Non-agricultural income	0.268	0.038	0.475	0.475
	(0.422)	(0.624)	(0.926)	(0.513)
Number of Labor force	−0.099	−0.014	−0.261	−0.261
	(0.063)	(−1.641)	(0.186)	(−1.398)
Proportion of migrant workers	0.025	0.004	0.115	0.115
	(0.240)	(0.105)	(0.582)	(0.198)
Per land area	−0.283 *	−0.040 *	−0.929 ***	−0.929 ***
	(0.161)	(−1.744)	(0.352)	(−2.639)
Distance	0.002	0.000	0.013	0.013
	(0.033)	(0.063)	(0.100)	(0.135)
Insurance	−0.171	−0.024	−0.498	−0.498
	(0.190)	(−0.876)	(0.540)	(−0.923)
Village terrain	1.218 ***	0.174 ***	0.174 ***	2.881 ***
	(0.236)	(4.764)	(4.764)	(4.926)
Generational differences_ middle-aged generation	−0.213	−0.030	−0.708	−0.708
	(0.465)	(−0.458)	(1.117)	(−0.634)
Generational difference_the older generation	−0.076	−0.011	−0.747	−0.747
	(0.653)	(−0.117)	(1.501)	(−0.498)
Constant term	−2.039		−5.030	
	(1.444)		(3.902)	
Whether control counties	Yes	Yes	Yes	Yes
N	540	540	540	540
Chi2	209.566			
P	0.000		0.000	
Pseudo *R^2^*	0.248		0.166	

Note: *** *p* < 0.01; ** *p* < 0.05; * *p* < 0.1; The numbers in parentheses under coefficient *B* are robust standard errors; *dy*/*dx* is the average marginal effect of the variable, and the numbers in parentheses are the Z-statistics.

**Table 3:** Robustness test of the influence of land attachment on land abandonment.

Variables	Model Replacement: Probit for Logit	Model Replacement: Tobit to Reg
	Abandonment Behavior	Abandonment Scale
Satisfaction	−0.430 **	−0.080 **
	(0.203)	(0.038)
Embeddedness	−0.317 **	−0.059 **
	(0.148)	(0.024)
Land dependence	0.103	0.008
	(0.151)	(0.029)
Control variables	Yes	Yes
Constant term	−4.218	0.066
	(3.033)	(0.519)
N	540	540
P	0.000	0.002
Chi2	235.150	
Pseudo *R*^2^	0.251	

Note: ** *p* < 0.05; Numbers in parentheses are robust standard errors.

**Table 4:** Intergenerational differences in the influence of land attachment on land abandonment.

Variables	New Generation		Middle-Aged Generation		Older Generation	
	Abandonment Behavior	Abandonment Scale	Abandonment Behavior	Abandonment Scale	Abandonment Behavior	Abandonment Scale
Satisfaction	−0.508 **	−0.884 **	−0.090	−0.359	−0.145	−0.406
	(0.222)	(0.412)	(0.152)	(0.520)	(0.172)	(0.371)
Embeddedness	0.392	0.358	−0.091	−0.134	−0.325 **	−0.775 **
	(0.263)	(0.395)	(0.169)	(0.498)	(0.164)	(0.384)
Land dependence	−0.005	0.191	−0.100	−0.518	0.027	0.173
	(0.142)	(0.291)	(0.136)	(0.467)	(0.097)	(0.260)
Control variables	Yes	Yes	Yes	Yes	Yes	Yes
Constant	0.908	3.983	−6.565 ***	−18.478 ***	1.383	−1.465
	(3.471)	(6.743)	(2.168)	(5.733)	(2.283)	(5.784)
N	105	105	256	256	179	179
P	0.000	0.000	0.008	0.000	0.000	0.010
Chi2	837.666		28.226		37.839	
Pseudo *R^2^*	0.267	0.217	0.113	0.071	0.068	0.044

Note: *** *p* < 0.01; ** *p* < 0.05; Numbers in parentheses are robust standard errors.

## Data Availability

Not applicable.
